# Detecting Suicide Ideation in the Era of Social Media: The Population Neuroscience Perspective

**DOI:** 10.3389/fpsyt.2022.652167

**Published:** 2022-04-14

**Authors:** Rosalba Morese, Oliver Gruebner, Martin Sykora, Suzanne Elayan, Marta Fadda, Emiliano Albanese

**Affiliations:** ^1^Faculty of Biomedical Sciences, Università della Svizzera italiana, Lugano, Switzerland; ^2^Faculty of Communication, Culture and Society, Università della Svizzera italiana, Lugano, Switzerland; ^3^Department of Epidemiology, Epidemiology, Biostatistics and Prevention Institute, University of Zurich, Zürich, Switzerland; ^4^Department of Geography, University of Zurich, Zürich, Switzerland; ^5^Centre for Information Management (CIM), School of Business and Economics, Loughborough University, Loughborough, United Kingdom

**Keywords:** suicide ideation, social media, epidemiology, neuroscience, mental health, Population Neuroscience, geography

## Abstract

Social media platforms are increasingly used across many population groups not only to communicate and consume information, but also to express symptoms of psychological distress and suicidal thoughts. The detection of suicidal ideation (SI) can contribute to suicide prevention. Twitter data suggesting SI have been associated with negative emotions (e.g., shame, sadness) and a number of geographical and ecological variables (e.g., geographic location, environmental stress). Other important research contributions on SI come from studies in neuroscience. To date, very few research studies have been conducted that combine different disciplines (epidemiology, health geography, neurosciences, psychology, and social media big data science), to build innovative research directions on this topic. This article aims to offer a new interdisciplinary perspective, that is, a Population Neuroscience perspective on SI in order to highlight new ways in which multiple scientific fields interact to successfully investigate emotions and stress in social media to detect SI in the population. We argue that a Population Neuroscience perspective may help to better understand the mechanisms underpinning SI and to promote more effective strategies to prevent suicide timely and at scale.

## Introduction

Worldwide, 800,000 people die by suicide every year, making suicide a public health priority (1, 2). The suicide rate increases during adolescence, and keeps growing in early adulthood and late life (3–5). A key aspect of prevention is addressing suicidal thoughts before they become an accomplished act (6). Suicidal ideation (SI) is usually defined as desire for death, thoughts about dying, and/or plans for suicide (7–9). However, these thoughts are not often and directly expressed, and are thus difficult to detect and to measure.

More than half of the global population (62.5%) has now access to the internet (10). While internet users spend 6.5 h online each day on average, social media platforms are widely used in the adult population, with 58% (4.62 billion) of the global population now being active social media users worldwide (10). Indeed, social media platforms are profoundly reshaping social life and social interactions and have a remarkable impact on how people communicate with each other (11). Psychological distress related to subjectively perceived stressful life events and to circumstances that may contribute to suicidal ideation and to suicide are increasingly expressed on social media such as Twitter, Reddit, or Instagram (12–15). The proportion of Twitter users who posted messages that indicate suicide risk factors have been correlated to suicide rates (16).

By applying epidemiological approaches, Twitter messages have been used to identify risk factors and a range of geographic and ecological variables (e.g., geographic location, environmental stressors) related to negative emotions, stress, and suicide (16–20). Another important approach to this branch of research comes from neuroscience that investigates the brain mechanisms underlying the cognitive and emotional processes involved in SI.

Population Neuroscience, in which epidemiology and neuroscience are combined to exploit their methodological strengths (21), represents a new research framework that “*emphasizes an understanding of human behavior across multiple levels of influence (e.g., from culture to social structure, experience, behavior, genes, neural connectivity, and function guided by a multilevel ecological model)*” (22). Also, other scholars highlighted that a Population Neuroscience perspective would help to better understand human behavior evolution and mental wellbeing across research disciplines (23). For example, Paus (24, 25) argues that the Population Neuroscience perspective will allow us to answer a new range of research questions through the combination of different methodologies and to reach new challenges in research between depth and breadth, with the combined neuroscience and epidemiology approaches, respectively. Paus also stresses that new knowledge will become available through the application of the Population Neuroscience approach and highlights new applications in preventive medicine, for example.

We argue that the Population Neuroscience perspective could be additionally promising in SI research, when combined with large scale social media analysis. While epidemiology focuses on the distribution and causes of disease and potential interventions to prevent disease and improve health in the population, neuroscience studies the brain, its mechanisms, and its relationship with mind and behavior. Both confer crucial knowledge on different levels, respectively at the population and individual level. To date, very few studies (26, 27) combined these different disciplines in the field of population mental health and rarely any study has used a Population Neuroscience approach combined with large scale social media data to study SI in the population.

Our aim was (1) to describe the combined role of epidemiology in the study of population risk factors and neuroscience by deepening the individuality of brain mechanisms, and (2) to explore the potential advantages offered by this interdisciplinary approach in the context of big social media data. We maintain that this perspective could inform the design of programs and interventions to prevent suicide.

### SI Studied Through Social Media Streams of Users

Wang et al. (28) pointed out that traditional methods such as clinical interviews or administering questionnaires have the disadvantage of not identifying suicidal thoughts in time. In contrast, social media can provide data that can be analyzed in real time (28). Some studies suggested that suicidal conversations on Twitter may be related to suicide (16, 29–32). Jashinsky et al. (16) filtered over 1.6 million tweets on Twitter using keywords and phrases generated from suicide risk factors for the United States and compared these with suicide rates. Results showed a strong correlation between United States per-state suicide data and Twitter conversations. O'Dea et al. (33) demonstrated that it is possible to identify Twitter messages related to SI by applying two different methods: human coders and automated machine classifiers. They applied words or phrases used by Jashinsky et al. (16) semantically and lexically consistent with SI. Nonetheless, according to the authors, an important limitation of their study was the lack of contextual information, and consequently the difficulty of differentiating between the messages of those who only expressed SI and those who were about to commit suicide (33).

In general, social networks are becoming a particularly relevant research area for suicide prevention, and approaches in this field open new methodological avenues. For example, Vioulès et al. (34) based their study on characteristics identified by the American Foundation for Suicide Prevention (AFSP) for three major individual level risk factors: (1) mental health (2) stressful conditions and adverse life events, and (3) previous suicide attempts and family history. For their study, the authors detected Twitter posts that included relevant key phrases created from two different lists, the American Psychiatric Association (APA) risk factors and the American Association of Suicidology (AAS) suicide warning signs. They concluded that by including not only the content of the messages but also the analysis of distress variables from these lists, it is possible to better discriminate against SI tweets (34). Coppersmith et al. (35) analyzed the messages and emotions posted by Twitter users before a suicide attempt. Their main results indicate that in the weeks preceding a suicide attempt, there is an increase in the expression of sadness. Conversely, they found that in the weeks following the suicide attempt, there is an increase in the expression of anger. This suggests that a longitudinal design is needed to analyze the emotions expressed in social media messages for SI.

This digital epidemiological approach (36) to assess SI risk in the population can be further expanded, integrating spatial epidemiology. For example, studies explored the use of geo-referenced social media data to identify emotional responses before, during, and after natural disasters and pandemics (18, 19, 37, 38) and in the context of a human made disaster (39). In the context of SI, an approach that integrates digital and spatial epidemiology can help in identifying geographic areas of increased SI risk in the population over time and allows exploration of locally specific exposure factors to SI. In turn, this information can inform the location and the design of tailored interventions to prevent suicide in specific regions and populations.

### The Neuroscience of SI

SI has been studied for years by different disciplines such as psychology and psychiatry, and more recently, SI has been increasingly studied in the field of neuroscience (7, 40–42). In the last decade, neuroscience has contributed to the understanding and identification of brain alterations that can contribute to SI and suicidal acts. Schmaal et al. (43) recently reviewed neuroimaging findings reporting the functional and structural neural circuits associated with SI and suicidal behavior. They identified studies that used neuroimaging methodologies such as functional magnetic resonance imaging (fMRI) in combination with words associated with suicide. Participants of these studies reported symptoms in line with suicide risk factors such as major depressive disorder, mood disorders, substance use disorders, schizophrenia, posttraumatic stress disorder, borderline personality disorder, bipolar disorder, and anxiety disorders (43). The majority of studies were conducted in adults, and only a small proportion examined adolescents, most likely for ethical reasons. Some studies suggested relationships between risk factors for SI, such as emotion dysregulation, anhedonia, impulsiveness, and brain areas (43–47). For example, specific brain areas such as the ventral prefrontal cortex (VPFC) and the dorsal prefrontal cortex (DPFC) were involved in the regulation of emotions (48) and impulses also in psychopathological disorders (49–52) with abnormalities in the prefrontal cortical region and lower neuronal density in ventral and dorsal regions found in the brains of those who have committed suicide (53, 54). The findings of Schmaal et al. (43) suggested that alterations in the medial and lateral VPFC regions may play a central role in the regulation of excessively negative and positive internal states that may influence SI. Scholarly work by Morese and Longobardi (7) and Longobardi et al. (55) also confirm that changes in the medial and lateral VPFC areas play a crucial role in SI and found that impairments of the cerebral system that involve the inferior frontal gyrus (IFG) and DPFC implicated in the control functions may favor suicidal behavior. Consequently, the combination of the deficits of both the VPFC and DPFC systems may represent a high-risk factor in which SI can be converted into lethal actions.

Examples exist to show how data and research from social media can be combined with neuroscientific knowledge. Online digital platforms, such as Facebook, Twitter, Reddit, or TikTok have been introduced as digital places where people can meet basic social needs to connect, interact, and communicate with others (56). Meshi et al. (57) suggest that these needs engage several specific neural systems in the brain. They propose the use of social media data alongside neuroscience to study similarities and dissimilarities between online and offline behaviors, especially social-cognitive processes in individual patients (57). An example of this may be examining friends and acquaintances networks on social media platforms and comparing their online behaviors in terms of similarities or dissimilarities to those of the real world. Emotions, thoughts, and intentions expressed on social media within these groups can be insightful when combined with inputs from neuroscience to predict real world behaviors of the studied individuals (57). Furthermore, social media metrics confer important advantages over other types of measures of people's social behavior (57). For example, according to Meshi et al. (57), social media data provides information and measures with high ecological validity by reporting indicators of people's behavior as they interact in the real world. These measures can be associated to people's behaviors to better prevent suicide. In addition, Yoo et al. (58) propose to develop a set of designs for clinician implications, on how to infer behavioral, cognitive, and social information from patients' social media data. This information can represent a “diary” of the patient's behaviors and could substantially enrich neuroscientific studies with variables of qualitative insights on patients.

### The Population Neuroscience Perspective

Population Neuroscience is an emerging and interdisciplinary field that combines neuroscience and epidemiology shifting the focus from individuals to population groups (21, 24, 59). The aim of Population Neuroscience is to pinpoint the factors that affect the health and wellbeing of the human brain during the life cycle by investigating large populations utilizing approaches from the fields of epidemiology, data science, genetics, and neuroscience with the investigation of genes and their regulation, external and internal environmental factors, and neural mechanisms, respectively (22, 24). To date, there are very few examples of empirical applications of this approach. Pan et al. (26) in a resting-state fMRI study reported that aberrant connectivity in the ventral striatum (i.e., a brain area involved in the reward system) may increase the risk of depressive disorder. Cirillo et al. (59) reviewed the scientific evidence and concluded that conducting longitudinal cohort studies in mental health research using a Population Neuroscience strategy is possible.

**Figure 1:**
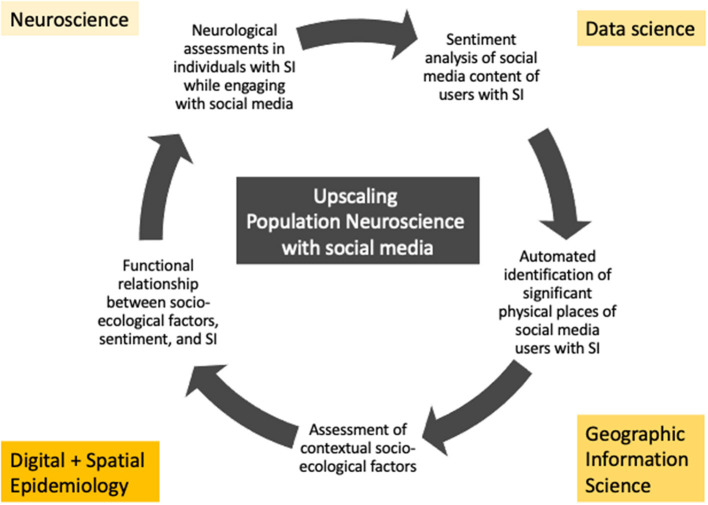
Upscaling population neuroscience approaches with social media: a generic work flow example.

We maintain that SI research will benefit from a Population Neuroscience perspective for at least five *reasons* and propose an application with social media in this context. *First*, neuroscientific studies have provided evidence for the role of some brain areas (e.g., VPFC and DPFC) involved during SI (43). However, in neuroscientific studies using structural magnetic resonance and fMRI brain studies, the number of participants scanned is usually low (60). Small sample sizes limit replicability and the external validity of findings (61–63). This limitation requires an increase in scale to detect and better understand the neural mechanisms of mental disorders in general and suicidal ideation in particular. A digital epidemiological approach using a combination of neuroscience and epidemiological methods and study designs, in population-based, large, and fairly representative samples can help overcome this limitation, for example using social media to facilitate participant recruitment into large-scale. The role of brain areas involved during SI can be intensively studied in combination with big social media data and the language used in text, audio, or video messages in selected individuals. As such, emotional stress can be analyzed in social media texts in conjunction with neurophysiological methodologies in people who tried to commit suicide, for example, linking the texts of those social media user timelines to MRI scans (Figure 1). Big social media data could then be used to scale up these linkages across large populations by investigating social media timelines of general users to detect individuals and population groups at risk for suicide.

**Figure 2:**
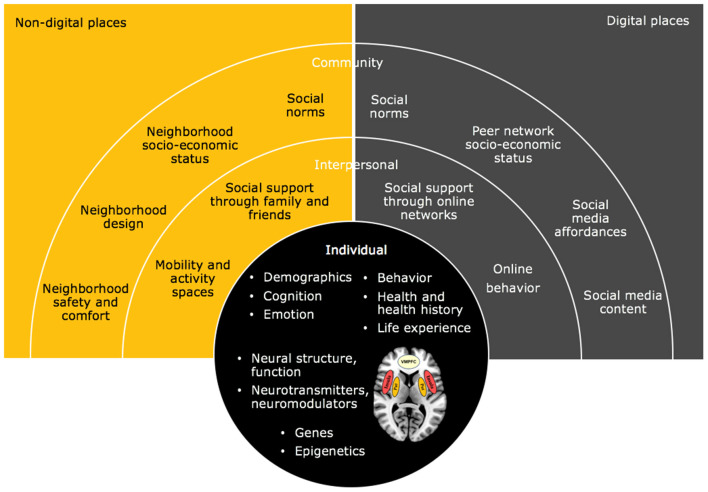
A representation of the population neuroscience perspective for detecting suicidal thoughts using a combined approach between neuroscience and epidemiology in the context of social media based on the social ecological model (22, 64, 65). The brain areas were processed for illustrative purposes using the MRIcron software package (65) on a standard T1 model and drawn based on a recent review (43) on suicidal thoughts and behaviors. VMPFC, ventral medial prefrontal cortex; Amy, amygdala; Put, putamen.

*Second*, linking digital (online) to non-digital (offline) places seems possible (56) (Figure 2). Analyzing the digital traces produced by social media users over time may provide contextual information on the non-digital places in which users live, work, or travel. For example, geo-referenced Twitter posts can be analyzed to identify places of significance to individual users (home, employment locations) (66). Such knowledge can be helpful to disentangle the effects of socio-ecological factors (air, noise, water pollution, poverty, crime) on SI and neural functioning at different places such as at home, at work, or on the daily commute. As such, evidence for locally specific exposure and resilience factors may be found in an Ecological Momentary Assessment (EMA) (67). Furthermore, arguably an EMA approach in such a setting may allow for analyzing users' mobility patterns as consequences of lockdowns or other restrictions implied by authorities to contain a pandemic disease, and in their functional relationship with mental health outcomes and brain activity, in line with Seidel et al's. (68) EMA and fMRI research examining emotions and anorexia.

*Third*, the providers of digital platforms have strong commercial interests and therefore develop features for their users that aim to command the greatest possible attention and attachment to keep users on their platforms (69). As such, it is important to also investigate the potentially multiple influencing factors of digital places and how these relate to brain activity. For example, the “#StatusOfMind” report found for the UK context, that while 91% of young adults (age 16–24 years) use social media, these forms of communication are considered more addictive than cigarettes and alcohol and that 7 in 10 young people experience cyberbullying (70). Negative effects of social media use on youth health further include symptoms of anxiety, depression, and poor sleep, possibly due to the fear of missing out, (the so called “FoMo” syndrome) while seeing their friends constantly posting images or status updates of seemingly enjoyable lives (70). The report continues to describe a “compare and despair” attitude that may be promoted by these feelings as users compare the (curated) images and posts of their friends to their own lives. Consequently, social media is considered to produce unrealistic expectations and to create feelings of self-consciousness, low self-esteem, or perfectionism that in turn could provoke anxiety disorders, problems related to body image, cyberbullying, and possibly SI (71, 72). The UNICEF report on the “State of the World's Children” further identifies at least three possible harms related to social media, including exposure to content (e.g., sexually explicit or discriminating), contact with adult initiated activities (e.g., interaction with ideological persuasion or sexual harassment), and also conduct of hostile peer activities (e.g., cyberbullying or stalking) (73).

*Fourth*, applying a Population Neuroscience approach also offers the opportunity to analyze the known potential risk factors from digital and non-digital places at multiple levels of influence, including individual personal, interpersonal, and community levels (Figure 2). More specifically, by applying epidemiological methodologies, it is possible to identify risk factors related to SI such as anxiety (74), most frequent migration history and/or social situation information (75), alcohol consumption (5), or major depressive and mood disorders in individuals and population groups (43). Schmaal et al. (43) suggest relationships between risk factors for SI, such as emotion dysregulation, anhedonia, impulsiveness, and the alterations in the medial and lateral VPFC, brain area involved in the regulation of excessively negative and positive internal states that can influence SI (7). Applying brain imaging methods allow us to identify and to deepen the understanding of the neural mechanism of SI, so that also the relationship between risk factors and brain function in SI can be better understood. Brain scan findings along with genetic dispositions can be studied in their associations with the individual (e.g., demographics), interpersonal (e.g., social support), and community level (e.g., socio-economic status) factors from non-digital and digital places, to get a deep-rooted understanding about the drivers of SI.

*Fifth*, assessing social media data in this context across geographic space may help to identify areas, population groups, and individuals with high suicide risk at scale. Once suicide risk or risk factors have been detected, it is imperative that researchers, social psychologists, school teachers, and policy makers design, evaluate, and contribute to the implementation of interventions for suicide prevention that promote and maintain mental health and wellbeing. Interventions could include a chatbot or an automated messaging service that can reach out to individuals and population groups who used language containing SI on social media. Guided by locally specific evidence on SI risk factors, interventions could also involve targeted community action plans helping prevent suicide in identified areas (76). More research is needed in this domain to determine legal, ethical, and effective approaches to prevent suicide once ideation has been expressed on digital and non-digital platforms (59).

In summary, Population Neuroscience is characterized by principles and tools useful for the study of population mental health, opening up new avenues in research on SI in the era of social media and can help plan for effective prevention interventions at scale.

## Discussion

We presented a new point of view for research on SI applying a perspective from Population Neuroscience, in which the different disciplines, epidemiology and neuroscience converge in the context of big data analytics and social media. Following the results presented by Cirillo et al. (59), we believe that it is essential to use this innovative approach to mental health research, specifically SI, because risk factors are populational, while disease is an individual experience.

A detailed understanding of individuals who have gone through SI as reflected in their social media feeds and behaviors, and how such behaviors may further inform differences in functional and structural neural circuits associated with SI and suicidal behavior remains virtually unexplored (43). The integration between fairly small samples in Population Neuroscience and large-scale big data on study participant behaviors for instance, to allow such study is noteworthy. A rare initial example of similar work is by Birnbaum et al. (77), who used 52,815 Facebook posts of 51 participants to model the likelihood of schizophrenia spectrum disorder psychotic episode relapses. The authors found that by using Facebook activity alone it was possible to predict hospitalization over a year in advance of such events (77). In a similar study, Birnbaum et al. (78) investigated 105 individuals and their 405,523 Google search queries over a year (i.e., *via* patient data donation) to classify psychiatric hospitalizations, finding that search frequency and search times already showed substantial differences of behavior from healthy individuals. Hence integrating social media data from online platforms with clinical information could 1 day serve to inform clinical decision-making and inform clinical care, while studies integrating big data from these platforms hold tremendous promise for research, and as we argue here, for Population Neuroscience in particular. Given for example, the functional magnetic resonance imaging (MRI) studies (43) on words associated with suicide, such specific linguistic features could be used in lexicon or ontology systems to map out their use across the content of social media messages. There are naturally substantial ethical and privacy concerns, but as elaborated by Chancellor et al. (79) these need to and can be addressed carefully, in a balanced manner. Ernala et al. (80) points out current methodological challenges in studying mental health through social media, and their core recommendations poignantly point out the need for “*building and utilizing shared infrastructures for data collection, and data donation effort”* and the importance of “*harnessing [interdisciplinary] partnerships between computational and clinical researchers, and patients”*.

Hence, big social media data can augment neuroscience studies in important ways. For example, at the individual level, additional social media data can help discover correlations with neuroscientific studies. At the population level, social media data may help in applying insights from individual level correlates across populations and extrapolating neuroscientific findings at an ecological level across populations of interest.

Equivalent to Cirillo et al. (59), we consider that Population Neuroscience offers new challenges and opportunities for mental health research. The interdisciplinary approach through the interaction between epidemiology and neuroscience could contribute to the advancement of both fields, better identifying the risk factors at the population level and deepening our understanding of the neural mechanisms involved in suicidal ideation at the individual level. New standards are needed to link knowledge about functions and structure of the human brain in the context of social media, not only to detect SI but also to offer effective social support to those in need, in a timely manner (81). We presented a new point of view building upon existing knowledge (22, 24, 25, 59). Our hope is that it can offer a new perspective, whereby epidemiology and neuroscience come together to leverage big social media data and maximize their potential to observe complex mental health phenomena at the population level.

We believe that this application can contribute to the design and implementation of innovative studies at a time when social media are increasingly used and evolving. The analysis of posted messages based on risk factors can be applied in an EMA approach to detect suicidal thoughts in real time. The application of a Population Neuroscience perspective to the study of SI presents a promising opportunity for further research and practice in the field of mental health. This new perspective will invariably be confronted with challenges, but it could open new fields of research and facilitate new prevention programs and interventions to counter suicide based on rapidly available data.

## Data Availability Statement

The original contributions presented in the study are included in the article, further inquiries can be directed to the corresponding author.

## Author Contributions

RM conceived the content of the article. RM and OG wrote the manuscript. SE, MS, MF, and EA reviewed the manuscript. EA in the role of principal investigator supervised the article. All authors contributed to the article and approved the submitted version.

## Funding

This work has been funded by the Zurich Foundation under the title: New technologies to improve mental health of the population. Open access funding was provided by Università della Svizzera italiana.

## Conflict of Interest

The authors declare that the research was conducted in the absence of any commercial or financial relationships that could be construed as a potential conflict of interest.

## Publisher's Note

All claims expressed in this article are solely those of the authors and do not necessarily represent those of their affiliated organizations, or those of the publisher, the editors and the reviewers. Any product that may be evaluated in this article, or claim that may be made by its manufacturer, is not guaranteed or endorsed by the publisher.
