# Erratum: Detecting suicide ideation in the era of social media: The population neuroscience perspective

**DOI:** 10.3389/fpsyt.2023.1186968

**Published:** 2023-03-24

**Authors:** Frontiers Production Office

**Affiliations:** Frontiers Media SA, Lausanne, Switzerland

**Keywords:** suicide ideation, social media, epidemiology, neuroscience, mental health, population neuroscience, geography

An omission to the funding section of the original article was made in error. The following sentence has been added: “Open access funding was provided by Università della Svizzera italiana.”

The original article has been updated.

